# Polymorphisms in Inflammasome Genes and Risk of Coal Workers' Pneumoconiosis in a Chinese Population

**DOI:** 10.1371/journal.pone.0047949

**Published:** 2012-10-22

**Authors:** Xiaoming Ji, Zhiguo Hou, Ting Wang, Kexin Jin, Jingjing Fan, Chen Luo, Minjuan Chen, Ruhui Han, Chunhui Ni

**Affiliations:** Department of Occupational Medicine and Environmental Health, School of Public Health, Nanjing Medical University, Nanjing, China; National Cancer Institute, National Institutes of Health, United States of America

## Abstract

**Background:**

Coal workers' pneumoconiosis (CWP), resulting from the inhalation of silica-containing coal mine dust, is characterized by fibrosing nodular lesions that eventually develop into progressive pulmonary fibrosis. Recently, it has been hypothesized that inflammasomes could have a crucial role in the host response to silica and recent studies show that the inflammasome contributes to inflammation and pulmonary fibrosis. NLRP3, CARD8 are components of the NLRP3 inflammasome, which triggers caspase 1-mediated IL-1β and IL-18 release. In the present study, we investigated whether common single nucleotide polymorphisms (SNPs) in inflammasome genes are associated with CWP.

**Methods:**

We performed an association study analyzing 3 *NLRP3*, 1 *CARD8*, 1 *IL-1β*, 2 *IL-18* SNPs in a case-control study of 697 CWP and 694 controls. Genotyping was carried out by the TaqMan method.

**Results:**

The *NLRP3* rs1539019 TT genotype was associated with a significantly increased risk of CWP (adjusted odds ratio (OR) = 1.39, 95% confidence interval (CI) = 1.07–1.81), compared with the GG/GT genotype, in particular among smokers (adjusted OR = 1.67, 95%CI = 1.15–2.42). In addition, the polymorphism was significantly associated with risk of CWP patients with stage I.

**Conclusions:**

This is the first report showing an association between the *NLRP3* rs1539019 polymorphism and CWP, and suggests that this polymorphism may confer increased risk for the development of the disease. Further studies are warranted to confirm our findings.

## Introduction

Coal workers' pneumoconiosis (CWP), which was originally thought to be a variant of silicosis, is one of the most widespread occupational lung diseases in China. It is a lethal fibrotic lung disease that results from the inhalation and retention of airborne coal mining dust which usually contains free crystalline silica within the lungs [1], characterized by lung chronic inflammation and fibrotic nodular lesions that usually leads to progressive fibrosis. Early pneumoconiosis can be asymptomatic, but advanced disease often leads to disability and premature death [2]. But there is no effective therapy for CWP, nor are the underlying immunologic mechanisms leading to disease clearly understood. Usually, the incidence and rate of CWP progression is related to the amount of respirable dust to which miners were exposed during their working lifetime. It is reported that the increasing prevalence and severity of coal workers' pneumoconiosis is due to increasing silica exposure in the United States [3]. In response to inhaled silica, alveolar macrophages and cytokines such as TGF-β1, interleukin (IL)-1β, IL-6 and IL-13 produced by these cells, have been suggested to play a central role during the early inflammatory response affecting the interactions among pro-and anti-inflammatory mechanisms that result in CWP [4]. Many factors attribute to CWP, including the workplace characteristics and susceptible individuals [5], [6]. Therefore, identification of new genetic factors for CWP, as well as safer work environment, is a need for strengthening CWP prevention measures.

Pathogen recognizing receptors (PRRs) are major triggers of innate immunity, including toll-like receptors and nod-like receptors (NLRs). Among the PRRs, the intracellular NLRs have recently been identified as key mediators of inflammatory and immune response [7], [8], [9], [10]. NLRP3 (NALP3/PYPAF1/Cryoprin/CIASI) belongs to the family of NLR proteins that comprise a nucleotide-binding domain and a leucine-rich repeat domain [11]. NLRP3 and CARD8 (also known as TUCAN) are constitutes of the inflammasome, which regulates IL-1β and IL-18 production [12]. The innate immunity can be activated through NLRP3 inflammasome sensing silica [13]. Stimulation of macrophages with silica leads to the activation of caspase-1 in an NLRP3-dependent manner but macrophages deficient in components of NLRP3 inflammasome were incapable of secreting the proinflammatory cytokines IL-1β and IL-18 in response to silica [14]. The activation of NLRP3 inflammasome may also result in the recruitment of fibroblasts and inflammatory cells and these cells play a pivotal role in fibrogenesis [15], [16]. CARD8 is a binding partner of the NLRP3 inflammasome and several studies have shown that it contributes to the development of some inflammatory diseases by acting as genetic susceptibility factors [17], [18], [19].

The involvement of inflammasome in silica recognition has been demonstrated [13], [14], [20]. Here we evaluated the frequency distribution of 7 common single nucleotide polymorphisms (SNPs) within 4 inflammasome genes (*NLRP3, CARD8, IL-1β, IL-18*) in CWP patients and control individuals, to investigate whether these SNPs could be associated with the susceptibility to CWP. *NLRP3* gene SNPs rs1539019 and rs4925648 were chosen since they have been previously associated with inflammatory disorders [21], [22], and the SNP rs10754558 in the 3′ untranslated region of *NLRP3* gene was selected because of the recently reported contribution to mRNA stability [23]. For *CARD8*, the C10X (rs2043211) polymorphism is a missense polymorphism, which is associated with inflammatory activity [18]. In addition, growing evidence suggests that variations of *IL-1β* and *IL-18* play a primary role in acute and chronic inflammation [24]. *IL-1β* rs16944, *IL-18* rs549908 and rs1946519 have been shown to be associated with susceptibility to chronic obstructive pulmonary disease [25], [26], pulmonary fibrosis [6], respectively. These variants may provide clues to the pathogenesis of CWP.

## Materials and Methods

### Study Population

Six hundred and ninety-seven CWP patients and 694 controls were recruited from the coal mines of Xuzhou Mining Business Group Co., Ltd. between January 2006 and December 2010, as described previously [27]. In brief, all subjects were underground coal miners and spent their entire working career within the above mentioned company. Therefore the dust exposure histories between cases and controls were comparable quantitatively and qualitatively. In addition, occupational health surveillance including physical examination and chest radiograph were taken every two years for all the underground coal miners, but it was not regular for the retirement. At the same time, high kilovolt chest X-rays were performed for reconfirming the diagnoses based on the China National Diagnostic Criteria for Pneumoconiosis (GBZ 70-2002), which is the same as the 1980 International Labor Organization (ILO) Classification of Pneumoconiosis in the judgment of opacity profusion [28]. Each case was classified as stage I, stage II and stage III according to the size, profusion and distribution range of opacities on chest X-ray by three national certified readers that required agreement at least of two readers. The controls were coal miners and matched with each cases for age (within 5 years), dust exposure period and job type. Each subject received an epidemiological questionnaire on individual information including age, respiratory symptoms, occupational histories, smoking habits and others. The questionnaire was done by face-to-face interviewers and blinded regarding the case or control status of participants. Blood sample of 5 ml was obtained from all subjects, and used for routine lab tests. This research protocol was specifically approved by the Institutional Review Board of Nanjing Medical University and all subjects gave their written informed consent before participating in the study.

### Genotyping

Genomic DNA was extracted from peripheral blood lymphocytes by the conventional phenol-chloroform method. Genotyping was performed using the TaqMan method with the ABI 7900HT Real Time PCR system according to the manufacturer's instructions (Applied Biosystem, Foster city, CA, USA) in a blinded fashion without knowledge of the workers' personal details or case status. The sequences of primer and probe for each SNP are available on request. Negative controls were included in each plate to ensure accuracy of the genotyping and approximately 10% of the samples were randomly selected for genotyping in duplicate to monitor genotyping quality and the results were 100% concordant. However, due to DNA quality several samples failed in genotyping, which were excluded in further analyses. For rs1539019, 8 DNA samples failed genotyping, including 4 CWP patients and 4 controls. For rs10754558, 6 cases and 3 controls DNA samples failed genotyping. For rs4925648, 9 cases and 7 controls DNA samples failed genotyping. For rs2043211, 9 cases and 4 controls DNA samples failed genotyping. For rs16944, 3 cases and 1 controls DNA samples failed genotyping. For rs549908, 4 cases and 7 controls DNA samples failed genotyping. For rs1946519, no DNA samples failed genotyping.

### Statistical analysis

Differences in the distributions of demographic characteristics, selected variables, and frequencies of genotypes between the CWP cases and controls were evaluated by using the Student's t-test or χ^2^-test. Hardy-Weinberg equilibrium (HWE) was tested using a goodness-of-fit χ^2^-test. The associations between genotypes and CWP were estimated by computing odds ratios (ORs) and their 95% confidence intervals (CIs) from unconditional logistic regression analysis with the adjustment for possible confounders. The statistical power was calculated by using the PS software (http://biostat.mc.vanderbilt.edu/twiki/bin/view/Main/PowerSampleSize). In this study, the dust-exposure cutoff used for the stratified analysis was according to the median of dust-exposure year of the recruited patients and controls. All statistical tests were two-sided at a significance level of 0.05 and were analyzed using the SAS software (version 9.1; SAS Institute, Inc., Cary, NC). The linkage disequilibrium was analyzed by the SHEsis software.

## Results

Seven inflammasome SNPs were genotyped in 697 CWP patients and 694 controls. The frequency distributions of the selected characteristics of the cases and controls are presented in Table 1. There was no significant difference in the distribution of age (*P* = 0.103), exposure years (*P* = 0.105), and work types (*P* = 0.534) between the cases and controls. The smoking status of CWP was similar to the controls (*P* = 0.250), but the pack-years smoked in CWP cases was significantly less than that of controls (*P*<0.001). The frequency distributions and means of the selected characteristics were matched adequately between cases and controls. In addition, of the 697 CWP cases, 415 (59.5%) were stage I, 219 (31.4%) were stage II and the remaining 63 (9.0%) were stage III.

**Table 1:** Demographic and selected variables among the CWP cases and control subjects.

Variables	CWP (*n* = 697)		Controls (*n* = 694)		*P*
	N	%	N	%	
Age, year (mean ± SD)	68.0±11.1		67.1±8.4		0.103
Exposure years (mean ± SD)	26.6±9.0		27.3±7.8		0.105
Smoking status					
Never	340	48.8	360	51.9	0.250
Ever	357	51.2	334	48.1	
Former	163	23.4	91	13.1	
Current	194	27.8	243	35.0	
Pack-years smoked					<0.001
0	340	49.2	360	52.6	
0–20	223	32.0	132	19.0	
>20	134	19.2	202	29.1	
Work type					0.534
Tunnel and coal mining	663	95.1	652	94.0	
Transport	16	2.3	17	2.5	
Others	18	2.6	25	3.6	
Stage					
I	415	59.5			
II	219	31.4			
III	63	9.0			

The primary information and allele frequencies observed are listed in Table 2. All genotyped distributions of control subjects were consistent with those expected from the Hardy-Weinberg equilibrium. The minor allele frequency (MAF) of all the 7 SNPs was consistent with that reported in the HapMap database (http://www.hapmap.org).

**Table 2:** Primary information of genotyped SNPs.

SNP	rs no.	Location	Base	MAF		HWE^a^
				Case	Control	
*NLRP3*	rs1539019	Intron	G>T	0.483	0.433	0.479
*NLRP3*	rs10754558	3′ UTR	C>G	0.475	0.469	0.656
*NLRP3*	rs4925648	Intron	C>T	0.237	0.234	0.241
*CARD8*	rs2043211	Exon 3	A>T	0.492	0.474	0.283
*IL1B*	rs16944	Promoter	G>A	0.495	0.465	0.550
*IL18*	rs549908	Promoter	T>G	0.136	0.111	0.842
*IL18*	rs1946519	Promoter	A>C	0.488	0.497	0.495

a HWE *P* value in the control group.

When considering SNPs in the inflammasome genes, *IL-1β* and *IL-18* polymorphisms were not associated with CWP (Table 3). *CARD8* rs2043211 polymorphism allelic and genotypes frequencies were similar in cases and controls. *NLRP3* rs10754558 and rs4925648 polymorphisms seem not be associated with CWP, whereas the rs1539019 minor T allele was significantly more frequent in patients than in controls (0.483 versus 0.433, *P* = 0.009) suggesting an increased risk of CWP (OR = 1.22). The genotype frequencies of *NLRP3* rs1539019 polymorphism (Table 3) were significantly different between the cases and controls (*P* = 0.028 and 0.009 for genotype and allele, respectively). However, this significance disappeared after the Bonferroni correction. With the current sample size an OR of 1.24 or higher and 0.80 or lower with an exposure frequency of 43% was detected with 80% power at significance level 0.05. Multivariate logistic regression analyses revealed that a significantly increased risk was associated with the TT genotype (adjusted OR = 1.53, 95%CI = 1.12-2.07), compared with the GG genotype.

**Table 3:** Distributions of genotypes of inflammasome genes and their associations with risk of CWP.

Variables	CWP cases		Controls		*P* a	OR (95% CI)	OR (95% CI)^b^
	N	%	N	%			
*NLRP3*(rs1539019)	*n* = 693		*n* = 690				
GG	186	26.8	217	31.5	0.028	1.00	1.00
GT	345	49.8	348	50.4		1.16 (0.90–1.48)	1.16 (0.91–1.48)
TT	162	23.4	125	18.1		1.51 (1.12–2.05)	1.53 (1.12–2.07)
G allele	717	51.7	782	56.7	0.009	1.00	
T allele	669	48.3	598	43.3		1.22 (1.05–1.42)	

*NLRP3*(rs10754558)	*n* = 691		*n* = 691				
CC	192	27.8	192	27.8	0.860	1.00	1.00
CG	342	49.5	350	50.6		0.98 (0.76–1.25)	0.98 (0.76–1.26)
GG	157	22.7	149	21.6		1.05 (0.78–1.42)	1.09 (0.81–1.47)
C allele	726	52.5	734	53.1	0.761	1.00	
G allele	656	47.5	648	46.9		1.02 (0.88–1.19)	

*NLRP3*(rs4925648)	*n* = 688		*n* = 687				
CC	401	58.3	409	59.5	0.732	1.00	1.00
CT	248	36.0	235	34.2		1.08 (0.86–1.35)	1.07 (0.85–1.34)
TT	39	5.7	43	6.3		0.93 (0.59–1.46)	0.90 (0.57–1.43)
C allele	1050	76.3	1053	76.6	0.839	1.00	
T allele	326	23.7	321	23.4		1.02 (0.85–1.21)	

*CARD8*(rs2043211)	*n* = 688		*n* = 690				
AA	173	25.2	162	23.5	0.094	1.00	1.00
AT	353	51.3	330	47.8		1.00 (0.77–1.30)	1.01 (0.78–1.31)
TT	162	23.5	198	28.7		0.76 (0.57–1.03)	0.78 (0.58–1.05)
A allele	699	50.8	654	47.4	0.074W	1.00	
T allele	677	49.2	726	52.6		0.87 (0.75–1.01)	

*IL1B*(rs16944)	*n* = 694		*n* = 693				
GG	172	24.8	202	29.2	0.186	1.00	1.00
GA	357	51.4	337	48.6		1.24 (0.97–1.60)	1.27 (0.98–1.63)
AA	165	23.8	154	22.2		1.25 (0.93–1.70)	1.30 (0.96–1.76)
G allele	701	50.5	741	53.5	0.119	1.00	
A allele	687	49.5	645	46.5		1.13 (0.97–1.31)	

*IL18*(rs549908)	*n* = 693		*n* = 687				
TT	520	75.0	542	78.9	0.132	1.00	1.00
TG	158	22.8	137	19.9		1.20 (0.93–1.56)	1.21 (0.93–1.57)
GG	15	2.2	8	1.2		1.95 (0.82–4.65)	1.88 (0.79–4.50)
T allele	1198	86.4	1221	88.9	0.051	1.00	
G allele	188	13.6	153	11.1		1.25 (1.00–1.57)	

*IL18*(rs1946519)	*n* = 697		*n* = 694				
AA	185	26.5	180	25.9	0.860	1.00	1.00
AC	344	49.4	338	48.7		0.99 (0.77–1.28)	1.00 (0.77–1.29)
CC	168	24.1	176	25.4		0.93 (0.69–1.25)	0.93 (0.69–1.25)
A allele	714	51.2	698	50.3	0.623	1.00	
C allele	680	48.8	690	49.7		0.96 (0.83–1.12)	

a Two-sided χ^2^ test.

b Adjusted for age, exposure years, and pack-years of smoking in logistic regression model.

In further stratification analysis for the SNP rs1539019 (Table 4), when we used the combined genotype GT/GG as the reference, we found that the TT genotype was associated with an increased risk of CWP (adjusted OR = 1.39, 95%CI = 1.07–1.81). This increased risk was also more pronounced among the subgroup of smokers (adjusted OR = 1.67, 95%CI = 1.15–2.42). Moreover, the polymorphism was significantly associated with risk of CWP patients with stage I. Additionally, significant associations were observed between the genotypes and patients with stage I (adjusted OR = 1.68, 95%CI = 1.25–2.26). However, no statistical evidence was found for the gene-environment interaction (data not shown).

**Table 4:** Stratification analyses between the genotypes of *NLRP3* rs1539019 and CWP risk.

Variables	Cases/controls	Genotypes (cases/controls)				*P* a	OR (95% CI)^a^
		GT/GG		TT			
		*n*	%	*n*	%		
Total	693/690	531/565	76.6/81.9	162/125	23.4/18.1	0.014	1.39 (1.07–1.81)
Exposure years							
<27	271/267	207/222	76.4/83.2	64/45	23.6/16.8	0.054	1.52 (0.99–2.33)
≥27	422/423	324/343	76.8/81.1	98/80	23.2/18.9	0.141	1.25 (0.92–1.80)
Smoking status							
Never	338/358	267/290	79.0/81.0	71/68	21.0/19.0	0.433	1.16 (0.80–1.69)
Ever	355/332	264/275	74.4/82.8	91/57	25.6/17.2	0.007	1.67 (1.15–2.42)
Stage							
I	413/690	302/473	73.1/68.6	111/217	26.9/31.4	0.0005	1.68 (1.25–2.26)
II	217/690	171/473	78.8/68.6	46/217	21.2/31.4	0.289	1.24 (0.84–1.83)
III	63/690	58/473	92.1/68.6	5/217	7.9/31.4	0.061	0.40 (0.16–1.04)

a Adjusted for age, exposure years, and pack-years of smoking in logistic regression model.

No linkage disequilibrium was found for three SNPs in *NLRP3* (rs1539019 and rs4925648: *r* ^2^ = 0.05, D′ = 0.42; rs1539019 and rs10754558: *r* ^2^ = 0.35, D′ = 0.61; rs4925648 and rs10754558: *r* ^2^ = 0.04, D′ = 0.37).

## Discussion

With respect to an association with risk of CWP in a Chinese population, we investigated seven polymorphisms in the inflammasome genes in our present study. We found that the *NLRP3* rs1539019 polymorphism was significantly associated with CWP, and the association was more evident in smokers. Furthermore, statistical evidence was observed for the polymorphism and CWP patients with stage I. To the best of our knowledge, this is the first study of the association of common SNPs in inflammasome genes with CWP risk.

CWP is a chronic inflammatory lung disease involving complex interactions among various environmental and genetic factors, although the pathophysiological mechanisms have not been fully identified. Genetic factors can modify the extent or severity of disease in susceptible individuals. Different genetic factors might be involved in the development of CWP [28], [29], [30], [31], and innate immune activation through NLRP3 inflammasomes sensing silica might be one of the immunologic mechanisms in CWP. Xu et al. indicated that the activation of NLRP3 played a potential role in the development of pulmonary fibrosis [32]. Several recent studies have shown that the NLRP3 inflammasome genes are associated with a number of autoimmune diseases including familial cold urticaria [33], Muckle-Wells syndrome [34] and multiple inflammatory diseases [35] such as Crohn's disease [36], obesity-induced inflammation and insulin resistance [37]. The activation of NLRP3 inflammosmes results in an inflammatory response mainly driven by the secretion of IL1β, a pro-fibrotic cytokine, playing an essential role in the pathogenesis of inflammation-induced pulmonary fibrosis [38]. Furthermore, NLRP3 inflammasome induces the MHC-II exposition on the macrophage/antigen presenting cells surface for a rapid non-self antigen presentation [39] and plays an important role in the maturation and activation of dendritic cells [40], [41], so the activation of NLRP3 drives the local inflammation as well as an acquired immune response. The pathogenesis of some of the most widespread pulmonary fibrotic diseases involves in the inflammatory and immune response [42]. Recently, a report demonstrated that missense mutations occurring in *NLRP3* enhanced the *NLRP3* mRNA stability [23]. Therefore, polymorphisms in NLRP3 inflammasomes could have a role in a not yet known pathologic defect and contribute to a CWP susceptibility genetic background. To date, there is no report on the association between the polymorphisms in NLRP3 inflammasome genes and risk of CWP.

In the present study, the most significant finding was the association between *NLRP3* rs1539019 and CWP risk. In the single-locus analysis, *NLRP3* rs1539019 TT genotype was associated with a significantly increased risk of CWP. The association was more pronounced between the polymorphism and CWP patients with stage I, suggesting that *NLRP3* rs1539019 may be involved in the development of CWP in the Chinese population. The possible explanation is that there may be different mechanisms underlying the early development of CWP and the subsequent progression of CWP [43], and the *NLRP3* rs1539019 may have different role in these two mechanisms. *NLRP3* rs1539019 is an intronic polymorphism whose function is less intuitive, whereas, recent studies have reported that intronic polymorphisms are associated with a variety of chronic diseases such as breast cancer [44], type II diabetes [45] and essential hypertension [46]. Euskirchen et al. showed that up to 40% of transcription factor binding sites were located within introns from immunoprecipitation and gene expression experiments [47]. Thus, the above results suggest that this may involve an area containing a regulatory sequence. When we use the MOTIF program (http://www.genome.jp/tools/motif/) to analyze the putative transcription factors for *NLRP3*, we found that the rs1539019 G>T locus is close to a 12-nucleotide sequence identified as the epidermal growth factor 1 consensus binding site, which is known to play a role in the vertebrate blood coagulation network [48]. Zhang et al showed that rs1539019 was one of the tag SNPs in the entire *NLRP3* gene, without significant association with major blunt trauma in Han Chinese population [49]. Omi et al reported that no statistically significant association between the rs1539019 polymorphism and essential hypertension risk was found in Japanese subjects [50]. Dehghan et al demonstrated that the rs1539019 polymorphism was associated with heart disease [22]. Consistently, *NLRP3* is strongly associated with the host immune response and susceptibility to inflammatory disorders [51]. In addition, the NLRP3 inflammasome is essential for the development of silicosis [14]. What has not been clear is the exact molecular mechanisms of how the rs1539019 polymorphism affect the risk of CWP, but it is possible that this tSNP may effect on the gene expression and that it may be in LD with other functional polymorphisms. However, these hypotheses need to be confirmed in further investigations.

In the present study, we also found that the increase risk associated with the rs1539019 TT genotype and was more evident among the smokers. The possible explanation is that individuals in this subgroup may be more likely to have been exposed to some risk factors involved in the etiology of CWP, such as tobacco smoking [52]. Several association studies have reported that smoking can induce lung fibrosis [53], [54].

In conclusion, our present study indicated that the *NLRP3* rs1539019 variant may confer increased risk of CWP in a Chinese population. To enhance the reliability of conclusions, further validation studies should strive to achieve diverse populations and larger sample size.
